# Mild hypothermia in combination with minimally invasive evacuation of hematoma reduces inflammatory damage in patients via the nuclear factor-κB pathway

**DOI:** 10.3892/etm.2014.2012

**Published:** 2014-10-09

**Authors:** YANPING BI, YING HUAN, WEIDONG CAI, XIA WANG, ZHIGANG LIANG, ZHAOKONG LIU, RUISHENG DUAN

**Affiliations:** 1 Department of Emergency, Shandong Provincial Qianfoshan Hospital, Shandong University, Jinan, Shandong 250014, P.R. China; 2 Department of Neurology, Shandong Provincial Jiaotong Hospital, Jinan, Shandong 250000, P.R. China; 3 Department of Maternal and Child Health Care, School of Public Health, Shandong University, Jinan, Shandong 250012, P.R. China; 4 Department of Neurology, Yantai Yuhuangding Hospital, Yantai, Shandong 264000, P.R. China; 5 Department of Neurology, Shandong Provincial Hospital, Jinan, Shandong 250021, P.R. China; 6 Department of Neurology, Shandong Provincial Qianfoshan Hospital, Shandong University, Jinan, Shandong 250014, P.R. China

**Keywords:** mild hypothermia, cerebral hemorrhage, minimally invasive hematoma evacuation, nuclear factor-κB

## Abstract

The aim of this study was to investigate the effects of mild hypothermia and minimally invasive evacuation of hematoma on the brain function of patients with cerebral hemorrhage. Seventy-six patients with acute cerebral hemorrhage were divided into the minimally invasive evacuation of hematoma (MIHE) and mild hypothermia and minimally invasive evacuation of hematoma (MHMIHE) groups. National Institutes of Health Stroke Scale (NIHSS) scores on the day of admission of the patient and one, three and seven days after the procedure were recorded. Perihematoma brain tissue morphology was observed using hematoxylin and eosin staining. Nuclear factor-κB (NF-κB) expression was determined by immunohistochemistry. The tumor necrosis factor-α (TNF-α) level was detected by ELISA. NIHSS scores in the MHMIHE group were significantly lower than those in the MIHE group on days three and seven. TNF-α and NF-κB levels peaked on day three, and the MHMIHE group had significantly lower levels of TNF-α and NF-κB than the MIHE group. In conclusion, the present study demonstrated that mild hypothermia and minimally invasive evacuation of hematoma can effectively reduce inflammation and improve the brain function of patients.

## Introduction

Cerebral hemorrhage, a common and frequently occurring disease with extremely high mortality and morbidity, accounts for 10–15% of all cerebrovascular strokes, causing a mortality rate that is >50% (1). Different treatment options exhibit different efficacies following cerebral hemorrhage. Minimally invasive hematoma evacuation following cerebral hemorrhage can reduce the hematoma-induced oppression of the surrounding tissues, release the ischemia and hydrocephalus caused by hematoma and extenuate perihematoma brain tissue damage aggravated by hematoma decomposition products, thus improving the brain function. In addition, mild hypothermia therapy exerts substantial protective effects on the brain (2,3), and has attracted considerable attention. This therapy can suppress the inflammatory response, reduce hydrocephalus and protect the brain. In recent years, the protection of the perihematoma brain tissue function has become a particular focus of studies of cerebral hemorrhage (4).

It is widely believed that the inflammatory response is involved in the pathological process of cerebral hemorrhage. In the early stage of cerebral hemorrhage, the local inflammatory response already exists in the tissues surrounding the hematoma, in which the inflammatory cytokine tumor necrosis factor-α (TNF-α) plays an important role. The aim of the present study was to enhance the understanding of the nuclear factor-κB (NF-κB) pathway-mediated inflammatory injury in perihematoma tissues. This was investigated using hematoxylin and eosin (HE) staining of perihematoma brain tissue slices and immunohistochemistry to examine the expression and distribution of NF-κB and peripheral vascular TNF-α following mild hypothermia in combination with minimally invasive evacuation of hematoma or minimally invasive evacuation of hematoma alone.

## Materials and methods

### Clinical data

In this study, 76 patients exhibiting the first onset of acute spontaneous intracerebral hemorrhage who were treated within 48 h of occurrence between September 2009 and September 2011 were selected (Table I). The study was approved by the Ethics Review Board of Shandong University (Jinan, China). Prior written informed consent was obtained from all the patients. The 76 patients were randomly assigned into two groups: The minimally invasive hematoma evacuation (MIHE) group, which contained 39 patients, and the mild hypothermia and minimally invasive evacuation of hematoma (MHMIHE) group, which contained 37 patients. All patients were confirmed for cerebral hemorrhage by computed tomography (CT) or magnetic resonance imaging. The volume of hemorrhage was >30 ml, as determined by CT film measurement and Tada formula (Volume = π × length × width × thickness/6) calculation (5). Prior and subsequent to treatment, the patients were all scored according to the National Institutes of Health Stroke Scale (NIHSS) with confirmation by the same neurologist prior and subsequent to scoring. All patients of the two groups were treated by minimally invasive hematoma evacuation on same or next day of the occurrence (<48 h). Patients of the MHMIHE group were additionally treated with mild hypothermia immediately following the surgery. Conventional treatments, including dehydrating agents and brain protection agents, were equally applied to the two groups. No statistically significant difference was identified between the two groups in gender, age, average initial NIHSS scores, position of hemorrhage and volume of hemorrhage. In order to avoid confounding factors for the inflammatory markers, the following situations were excluded: i) Inflammation from infections or nosocomial infections within the past two weeks; ii) severe diseases of the major organs, such as the heart, liver, lungs and kidney, as well as endocrine, blood, neoplastic and immune system diseases; iii) surgery, trauma, heart and brain strokes, and pregnancy within six months or at ages <18 years; iv) being treated with glucocorticoids, anticoagulants or β-receptor inhibitors; and v) reluctance exhibited by the patients or their family members for the surgery.

### Minimally invasive hematoma evacuation

Minimally invasive hematoma evacuation was performed under local anesthesia using the YL-1 disposable intracranial hematoma crushed puncture needle (Beijing WanTeFu Medical Apparatus Co., Ltd., Beijing, China).

### Mild hypothermia therapy

For body cooling, three patients were treated using an intravascular cooling instrument (CoolGard 3000^®^; Alsius, Chelmsford, MA, USA), while the remaining 34 patients were treated using body water circulation cooling blankets (Model P&C-A; Beijing Hengbang Kaijie Heating Radiator Co., Ltd., Beijing, China). For brain local mild hypothermia therapy, all the patients were treated using ice hats between −4 and +2°C (HGT-200; Beijing Dawei Tongchuang Medical Treatment Equipment Co., Ltd, Beijing, China). Patients with shivering were administered sedative drugs. Brain temperatures were reduced to 32.5–34.5°C, and were obtained by measuring the tympanic membrane temperatures on the same side of the nidus (brain temperature = tympanic membrane temperature ± 0.5°C) using an infrared ear thermometer (Omron Corp., Kyoto, Japan) twice a day and recording the higher temperature.

### NIHSS scoring

Prior and subsequent to treatment, the patients were scored according to the NIHSS (6,7), with the full score being 22. The degree of neurological deficit was classed as low, medium or high. NIHSS scores were defined as follows: Low, <7; medium, 7–15; and high, >15.

### HE staining

Samples of perihematoma brain tissue were fixed, embedded in paraffin and cut into tissue sections. Next, the tissue sections were dewaxed using xylene and rehydrated using graded alcohols. After washing with running water and distilled water, the sections were stained with hematoxylin for 3–5 min. Following further washing with running water, the sections were differentiated using 1% HCl in 70% alcohol. Subsequently, the sections were stained with eosin for 1–4 min, after washing with running water. Following dehydration and differentiation in alcohol, the sections were mounted and observed under an Olympus CX41 inverted fluorescence microscope (Olympus Optical Co., Ltd., Tokyo, Japan).

### Immunohistochemistry

Perihematoma brain tissue fragments from all patients were washed out during the surgery and at one, three and seven days after the surgery, fixed by formalin, and paraffin-embedded for slicing. Following slicing, the brain tissue slices were dewaxed by xylene, processed by ethanol and fixed by acetone. Endogenous peroxidase was then deactivated by 3 ml/l H_2_O_2_ for 10 min, and the slices were washed with double-distilled H_2_O and soaked with phosphate-buffered saline (PBS) for 5 min. Normal goat serum was subsequently added to block the tissue for 40 min. Following blocking, rabbit anti-human NF-κB (Santa Cruz Biotechnology, Inc., Santa Cruz, CA, USA) was added, and the slices were incubated overnight at 4°C and washed for 5 min with PBS three times. An equal amount of biotinylated goat anti-rabbit IgG (Abcam, Cambridge, MA, USA) was subsequently added, and the sections were further incubated for 30 min at 37°C and washed for 5 min with PBS four times. Following washing, avidin-biotin complex was added, and the slices were incubated for 30 min at 37°C and washed for 5 min with PBS four times. The samples were then dyed for 3 min by diaminobenzidine and the reaction was terminated by soaking with PBS, prior to the samples being prepared for optical inspections. Positive cells were dyed dark brown in the cytoplasm and nucleus. The number of positive cells was counted under the microscope (Olympus CX41 inverted fluorescence microscope), and the density of positive cells was calculated.

### Statistical analysis

The results were analyzed using SPSS 10.0 software (SPSS, Inc., Chicago, IL, USA). The counting and measurement data are presented as the mean ± standard deviation, while two groups of mean values were compared using an independent sample Student’s t-test. P<0.05 was considered to indicate a statistically significant difference.

## Results

### General patient data

In this study, 76 patients with the first onset of acute spontaneous intracerebral hemorrhage were enrolled. The 76 patients were randomly assigned into the MIHE (39 patients) and MHMIHE (37 patients) groups. Basic patient data on admission are provided in Table I. To evaluate the changes in neurological function damage, NIHSS scoring of patients in the two groups was performed. As shown in Table II, the scores of the two groups were the highest on admission and on the first day after treatment, and decreased gradually with the progression of treatment. No statistically significant differences were identified between the two groups (P>0.05) on admission and the first day after treatment. The scores of the MHMIHE group on the third and seventh day after treatment were lower than those of the MIHE group, with statistically significant difference (P<0.05). These results suggested that mild hypothermia and minimally invasive evacuation of hematoma reduces the damage to the neurological function of perihematoma brain tissues and protects the brain tissues.

### Dynamic changes in TNF-α levels in the serum of the two groups of patients

To assess whether mild hypothermia and minimally invasive evacuation of hematoma could alleviate inflammatory responses in the perihematoma brain tissues, the content of TNF-α in the serum of the two groups of patients was tested by ELISA assay. As shown in Table III, the content of TNF-α in serum of the two groups of patients was elevated on day 1 (MIHE, 3.0223±0.4799 ng/ml; MHMIHE, 2.9492±0.6069 ng/ml), reached peak value on day 3 (MIHE, 3.4363±0.6374 ng/ml; MHMIHE, 2.8180±0.2178 ng/ml) and showed the lowest value on day 7 (MIHE, 2.7354±0.8083 ng/ml; MHMIHE, 1.1560±0.7074 ng/ml). Of note, the content of TNF-α in the MHMIHE group at each time-point was lower than that of TNF-α in the MIHE group (P<0.05). The time course of these changes concurred with that observed in the HE staining and NIHSS scoring, as well as with that observed for the NF-κB analysis. These results suggested that mild hypothermia and minimally invasive evacuation of hematoma alleviated the inflammatory responses in the perihematoma brain tissues.

### Pathological observations of perihematoma brain tissues

**Figure 1:**
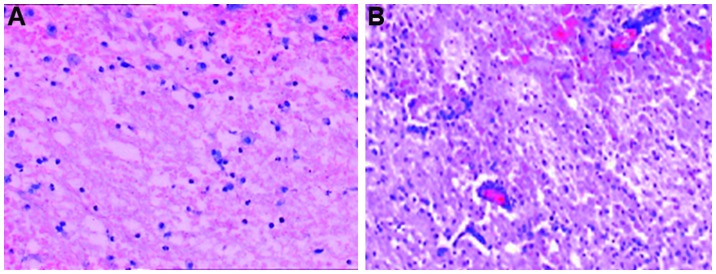
Hematoxylin and eosin staining (magnification, ×100). (A) Day 1 of patients following minimally invasive evacuation of hematoma. The perihematoma tissues were infiltrated by inflammatory cells and appeared to be loose. The extravascular space was expanded. (B) Day 1 of patients following mild hypothermia and minimally invasive evacuation of hematoma. Vascular congestion was observed in the tissues surrounding the hematoma in the acute phase. The size of nerve cells was reduced, karyopyknosis was observed, Nissl substance had disappeared and a large number of neutrophils and lymphocytes had infiltrated.

To investigate the pathological changes in the perihematoma brain tissues, fragments of perihematoma brain tissues were fixed, paraffin-embedded and sliced for HE staining. The histological analysis (Fig. 1) revealed that the perihematoma tissues were loose and the extravascular space was expanded in patients treated with minimally invasive evacuation of hematoma (Fig. 1A). However, space had appeared around the nerve and glial cells and the size of nerve cells was reduced, and karyopyknosis was observed in patients treated with mild hypothermia and minimally invasive evacuation of hematoma (Fig. 1B). Furthermore, in patients treated with mild hypothermia and minimally invasive evacuation of hematoma, Nissl substance had disappeared and the cytoplasm was observed to have developed eosinophilic changes. Large numbers of neutrophils and lymphocytes appeared around the hematoma. These results suggested that edema, necrosis and inflammatory responses occurred in the perihematoma brain tissues.

### Changes in NF-κB in the perihematoma brain tissues of the two groups of patients

**Figure 2:**
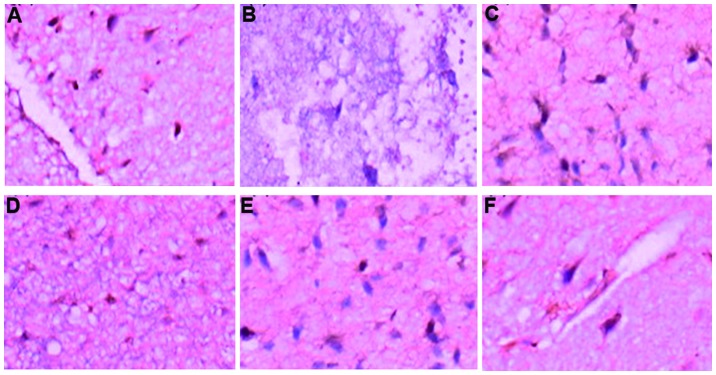
Immunohistochemical assays of nuclear factor-κB expression. (A) Group MIHE, day 1 (magnification, ×400); (B) Group MHMIHE, day 1 (magnification, ×200); (C) Group MIHE, day 3 (magnification, ×400); (D) Group MHMIHE, day 3 (magnification, ×200); (E) Group MIHE, day 7 (magnification, ×400); (F) Group MHMIHE, day 7 (magnification, ×400). MIHE, minimally invasive evacuation of hematoma; MHMIHE, mild hypothermia and minimally invasive evacuation of hematoma.

To assess whether mild hypothermia and minimally invasive evacuation of hematoma effectively reduced NF-κB levels in perihematoma brain tissues, immunohistochemical assays were performed. In the immunohistochemical staining (Fig. 2), NF-κB was expressed in the 76 patients with dynamic changes. NF-κB levels reached the peak on the third day and were elevated on the first and seventh days. The NF-κB expression was reduced for the MHMIHE group. The expression was mainly localized in inflammatory tissues, microglia and nerve cells. These results suggested that mild hypothermia and minimally invasive evacuation of hematoma effectively reduced the expression of NF-κB in perihematoma brain tissues and therefore alleviated inflammatory damage.

### CT of patient brains

**Figure 3:**
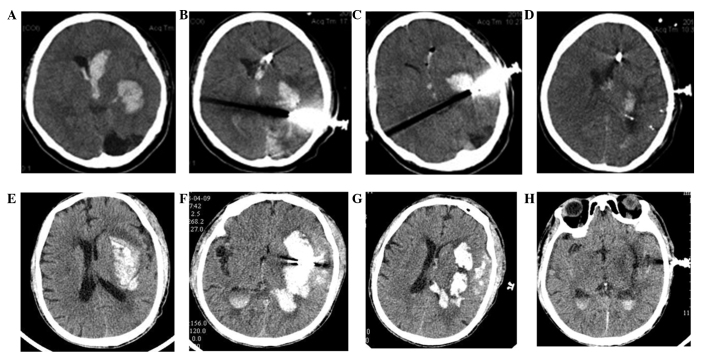
Brain CT film results of patients treated with (A–D) minimally invasive evacuation of hematoma 3 h after the onset, and (E–H) minimally invasive evacuation of hematoma and subsequent mild hypothermia 2 h after the onset. Brain CT film was obtained at (A) 3 h after the onset; (B) 1 day after surgery; (C) 3 days after surgery; (D) 7 days after surgery; (E) 2 h after the onset; (F) 1 day after surgery; (G) 3 days after surgery; and (H) 7 days after surgery. CT, computed tomography.

To compare the effects of the two types of treatments on the two groups of patients with cerebral hemorrhage, the brains of the patients were examined by CT on days 1, 3 and 7. As shown in the brain CT film (Fig. 3) on admission and following surgery, lateral ventricle and local hematoma was significantly reduced on the first day after surgery, and this change became more significant on the seventh day after surgery. These results suggested that mild hypothermia and minimally invasive evacuation of hematoma effectively alleviated cerebral hemorrhage and improved brain function.

## Discussion

The brain protection effect of mild hypothermia therapy was first applied by Busto in 1987 and has become a focus in brain protection studies in recent years (8). Studies have suggested that mild hypothermia therapy >48 h can effectively reduce the damage to the brain (6,7). However, the therapy should be kept <196 h at a temperature of 32–35°C. Cardiopulmonary resuscitation research found that patients could reach the target temperature (34°C) 2 h earlier if the temperature began to decrease at the beginning of resuscitation, with higher safety and feasibility, as well as improved prognosis of the nervous system (6,7,9). In addition, no additional arrhythmia, infection, coagulopathy or hypotension was observed. Therefore, whole body cooling and brain local mild hypothermia therapy was selected to reduce the brain temperature to 32.5–34.5°C. The results showed that the NIHSS scores of the degree of neurological deficit, the expression of NF-κB in perihematoma brain tissue and the concentration of TNF-α in serum in the MHMIHE group of patients were improved compared with those in the MIHE group. Therefore, patients may increasingly benefit from this advance in medical technology.

In the acute phase of cerebral hemorrhage, hematoma exerts oppression to its surrounding tissues, and blood, hemoglobin and their decomposition products enter the brain tissue, causing cerebral ischemia, hypoxia or even necrosis. This induces damage from the immune and inflammatory response and therefore generates a high levels of inflammatory factors (10). A positive correlation exists between the amount of bleeding and this damage (11,12). Minimally invasive evacuation of hematoma can reduce the size of the hematoma, release the hematoma oppression to the surrounding tissues, ease cerebral edema, lower the intracranial pressure, reduce the inflammatory factor production and inflammatory damage, and protect the brain function (13–16).

The present results showed that, following cerebral hemorrhage, the TNF-α concentration and NF-κB expression in the blood followed the same trend when the patient condition changed. The concentration of TNF-α in serum began to rise on the first day and reached the peak on the third day; the concentration on the seventh day was the lowest. Similar changes were observed for NF-κB expression. At each time-point, the TNF-α concentration and NF-κB expression of the MHMIHE group were lower than those of the MIHE group (P<0.05). The NIHSS scores of the neurological deficit degree of the two groups were the highest on admission and on the first day after treatment, with no statistically significant difference between the two groups (P>0.05), and decreased gradually with the progression of treatment. The score on the third day was higher than that on the seventh day, which was consistent with the changes in TNF-α and NF-κB expression. The degree of neurological deficit in the MHMIHE group on the third and seventh day was improved compared with that in the MIHE group, with a statistically significant difference (P<0.05). The NIHSS scores of the neurological deficit degree were lower in the MHMIHE group than those in the MIHE group, and no other complications, such as arrhythmia and infection, were observed. This result suggests that the NF-κB-mediated inflammatory response participated in the pathological process of brain tissue damage following cerebral hemorrhage. Mild hypothermia and minimally invasive evacuation of hematoma can alleviate the pathological damage of the brain tissue caused by the NF-κB-mediated inflammatory response. Similarly, the NF-κB expression, TNF-α level and NIHSS scores of the MHMIHE group were improved compared with those of the MIHE group (P<0.05).

NF-κB is a type of important transcription factor that participates in the regulation of mRNA transcription of multiple target genes, including TNF-α, and affects their protein synthesis (17). TNF-α is a key cytokine for the inflammatory response following brain damage, and plays an important role in the inflammatory damage caused by cerebral hemorrhage (18). Mild hypothermia can reduce vascular permeability, stabilize ion pumps, inhibit nerve excitability cascade reactions and lower the brain metabolism to suppress inflammation and immune responses, and therefore protects the brain function following cerebral hemorrhage (19).

The present study used minimally invasive evacuation of hematoma to alleviate the damage of the hematoma to the surrounding brain tissues. At the same time, in order to improve the brain function, mild hypothermia therapy was used to reduce the inflammation and immune responses that could cause damage to the brain tissues. The results demonstrated the effectiveness of the mild hypothermia and minimally invasive evacuation of hematoma procedure.

## Figures and Tables

**Table I:** Patient information.

	Group MIHE, n=39	Group MHMIHE, n=37	P-value
Age (years)	52.70±16.24	50.38±11.32	0.09
Admission delay (h)	5.42±1.85	4.63±2.46	0.11
NIHSS score	16.98±3.02	17.22±2.91	0.08
Bleeding (ml)	39.39±8.12	39.48±10.56	0.07
Systolic pressure (mmHg)	149.92±14.08	145.72±19.11	0.91
Diastolic pressure (mmHg)	95.58±12.25	87.25±10.65	0.89
Body temperature (°C)	37.28±0.48	37.03±0.42	0.31
Leukocytes (×10^9^)	12.05±2.74	11.86±1.81	0.29
Mononuclear cells (×10^9^)	0.78±0.34	0.74±0.48	0.23
Lymphocytes (×10^9^)	4.44±0.51	4.05±0.17	0.30
Neutrophils (×10^9^)	7.98±2.88	7.85±1.42	0.33
Blood sugar (mmol/l)	8.07±1.77	8.55±4.68	0.56

Data are presented as the mean ± standard deviation. MIHE, minimally invasive evacuation of hematoma; MHMIHE, mild hypothermia and minimally invasive evacuation of hematoma; NIHSS, National Institutes of Health Stroke Scale.

**Table II:** National Institutes of Health Stroke Scale scores of the two groups.

Group	n	Admission	Day 1	Day 3	Day 7
MIHE	39	16.98±3.02	16.48±3.02	15.98±2.69	14.42±1.23
MHMIHE	37	17.22±2.91	16.32±2.91	15.02±1.81	12.14±2.02

Data are presented as the mean ± standard deviation. P>0.05 between the two groups on admission and day 1 after treatment; P<0.05 between the two groups on days 3 and 7 after treatment. MIHE, minimally invasive evacuation of hematoma; MHMIHE, mild hypothermia and minimally invasive evacuation of hematoma.

**Table III:** Dynamic changes in tumor necrosis factor-α levels in the serum of the two groups.

Group	n	Day 1 (ng/ml)	Day 3 (ng/ml)	Day 7 (ng/ml)
MIHE	39	3.0223±0.4799	3.4363±0.6374	2.7354±0.8083
MHMIHE	37	2.9492±0.6069	2.8180±0.2178	1.1560±0.7074

Data are presented as the mean ± standard deviation. P<0.05 between the two groups at each time-point. MIHE, minimally invasive evacuation of hematoma; MHMIHE, mild hypothermia and minimally invasive evacuation of hematoma.
